# Use of artisanal simulators in the ultrasound training for invasive procedures in nephrology: venous access and renal biopsy

**DOI:** 10.1590/2175-8239-JBN-2018-0211

**Published:** 2019-06-27

**Authors:** Marcus Gomes Bastos, Ramon de Oliveira Dalamura, Ana Luisa Silveira Vieira, José Pazeli

**Affiliations:** 1 Universidade Federal de Juiz de Fora Faculdade de Medicina Programa de Pós-Graduação em Saúde Juiz de ForaMG Brasil Universidade Federal de Juiz de Fora, Faculdade de Medicina, Programa de Pós-Graduação em Saúde, Juiz de Fora, MG, Brasil.; 2 Fundação Governador Ozanam Coelho Faculdade de Medicina UbáMG Brasil Fundação Governador Ozanam Coelho, Faculdade de Medicina, Ubá, MG, Brasil.; 3 Fundação Instituto Mineiro de Estudos e Pesquisas em Nefrologia Juiz de ForaMG Brasil Fundação Instituto Mineiro de Estudos e Pesquisas em Nefrologia, Juiz de Fora, MG, Brasil.; 4 Faculdade de Medicina de Barbacena BarbacenaMG Brasil Faculdade de Medicina de Barbacena, Barbacena, MG, Brasil.; 5 Faculdade de Medicina SUPREMA Juiz de ForaMG Brasil Faculdade de Medicina SUPREMA, Juiz de Fora, MG, Brasil.

**Keywords:** High Fidelity Simulation Training, Ultrasonography, Image-Guided Biopsy, Vascular Access Devices, Nephrology

## Abstract

**Introduction:**

Vascular access and renal biopsy are common procedures in nephrology. In this study, two artisanal simulators of very low cost and excelent image quality are (prented) presented to guide, by ultrasound, the venous access and renal biopsy.

**Methods:**

The simulators are constructed using chicken breast slices, Penrose drain, plastic milk shake straw and pig kidney.

**Results:**

Both simulators enable immediate identification of the anatomical structures of interest, vessels and kidney, and enable spatial orientation and hand-eye coordination, essential for the development of the necessary skills to safely carry out invasive procedures.

**Conclusion:**

The simulators described, were extremely useful for simulating venous access and renal biopsy guided by ultrasonography, enabling training to reduce the failure rate in punctures and the potential complications associated with the described procedures.

## INTRODUCTION

Simulation is an effective pedagogical strategy in medicine and has gained momentum at different levels in healthcare education.^1^ For instance, an important step in the training of ultrasound-guided procedures is the development of dexterity in simultaneous manipulation of the ultrasound probe and the puncture-needle within a three-dimensional space from a two-dimensional image.^2^ In this sense, the use of realistic simulators has great potential to guarantee the development of skills to perform venous access (internal jugular, subclavian, femoral and arteriovenous fistula) and US-guided renal biopsy, frequent procedures in a nephrologist’s practice. In the present work, we describe the development of non-human artisanal simulators extremely easy to mount and of low coast, for the practical training of venous access and renal biopsy guided by ultrasonography.

## METHODS

**Figure 1:**
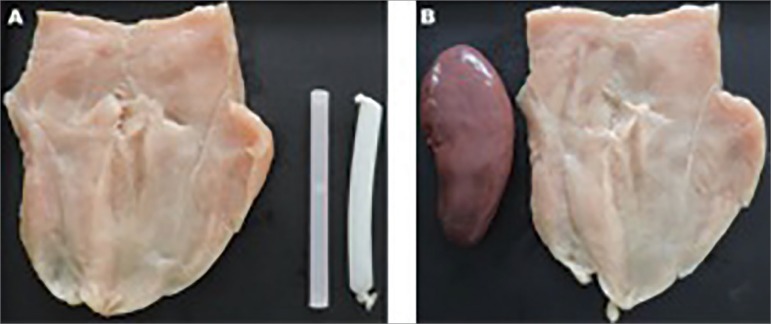
Material used in the construction of the homemade simulators from chicken breast. In A, we see a chicken breast slice, a Penrose drain and a milkshake straw, already filled with ultrasound gel. In B, we see a pig kidney and a slice of chicken breast.

Two artisanal simulators for vascular access and renal biopsy training were assembled from slices of chicken breast, pig kidney and other easily accessible and low cost materials. The vessel simulator was developed with a Penrose drain (number 2), a soft, flexible rubber tube, latex type used as a surgical drain, to prevent the buildup of fluid in a sugical site, and a milk shake plastic straw (10 mm diameter), stiffer plastic material, both filled with ultrasound gel (Figure 1A). Next, both “vessels” were placed parallel to each other, between two chicken breast slices of about 1 cm thick (approximately the depth of the internal jugular vein relative to the skin of an adult individual), and involved with transparent PVC film.

The artisanal simulator for renal biopsy was developed using pig kidney, dissected in order to remove perirenal fat and the renal capsule (Figure 1B). The kidney was then wrapped with layers of chicken breast (about 1.0 cm thick each), one below and two or three above, to simulate the depth of the adult kidney and finally wrapped with transparent PVC film.

Ultrasound images were obtained in B mode using high frequency 4 to 15 MHz (for “vascular” images) and low frequency of 2 to 5 MHz (in the simulation of renal biopsy), conected to uSmart ultrasound (Terason, USA).

## RESULTS

**Figure 2:**
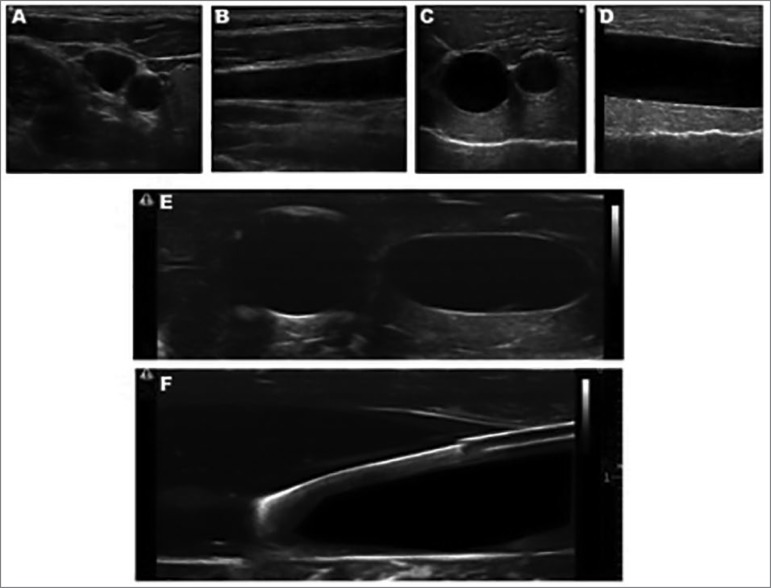
Homemade Simulator used to practice ultrasound-guided vascular Access. A and B, ultrasound images from the internal jugular vein and the carotid artery of one of the authors (RD); C and D, images generated from the homemade Simulator built from chicken breast; E, using the compression technique to differentiate the artery (on the left) from the vein (on the right); F, visualization of the guide-wire and the puncture needle inside the vein in the simulation.

The homemade simulator for the training of vascular access guided by ultrasonography enabled the identification and differentiation between the “vein”, represented by the Penrose drain and comprehensible to the probe pressure on the anterior surface of the simulator, and the “artery”, portrayed by the milk shake straw, resistant to compression (Figure 2). High quality imaging also enabled the identification and manipulation of the puncture needle under the insonation area of the ultrasound probe by different techniques (in the plane, out of plane and oblique) (Figure 2). Additionally, the filling of the "vessels" with ultrasound gel, a viscous material that does not leak after the puncture, allows multiple punctures of the Penrose drain. Also, the texture of chicken breast enables to follow the puncture needle from the surface of the phantom to the interior of the Penrose drain.

**Figure 3:**
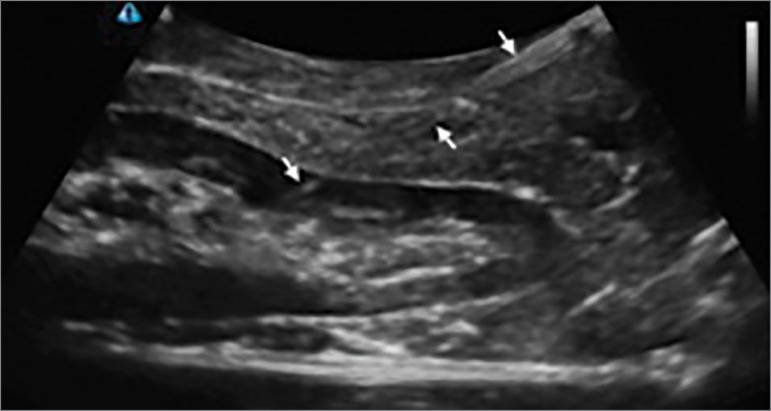
Homemade renal simulator with the biopsy needle (arrows) in its trajectory to the kidneys.

The kidney biopsy simulator made with chicken breast, of easy and quick assembly, mimics the acoustic properties of the tissues and represents the sonographic image of the kidney. It enables visualizing the biopsy needle in its path to the kidney (identified by the arrows in Figure 3), to train the “jabs” technique and practice harvesting renal tissue with a biopsy needle. In addition, the chicken breast is not damaged with the repeated passage of the needle, differenty what happens with commercially available simulators. The short shelf life and the strong odor exhaled by the chicken breast when kept at room temperature are factors that can be overcome by keeping the simulators in the refrigerator at low temperature (-20⁰C), for till three weeks. Finally, filling the Penrose drain with ultrasound gel, enables multiple punctures to be made, which is very important in training sessions involving multiple participants.

## DISCUSSION

Simulation is not a new strategy, it has been in use since 1929, when Ed Link developed a simulator to train airplane pilots.^3^ In healthcare, and simulation has gained space, particularly with regard to invasive medical procedures. For example, the use of ultrasonography before or during venous access allows to increase the rate of success of the first cannulation and reduction of complications.^4^^,^^5^

Technological advances have enabled the development of highly sophisticated and realistic phantoms, enabling the training of risky invasive procedures in low stress environments. However, these commercial phantoms are invariably costly and the material used is often damaged as the number of manipulations increases, particularly when using a needle.

In our study, we present two artisanal simulators for vascular access training and ultrasound-guided renal biopsy. The simulators were developed using chicken breast - a tissue that bears similar echogenicity to its human counterpart,^6^ a tissue showing similar echogenicity to human being counterpart, Penrose drain and a milk shake straw (to simulate blood vessels); and a pig kidney (for renal biopsy training), low cost and easily accessible materials. The assembly of both homemade simulators are very simple and quick, not exceeding five minutes for each one. In addition, the images obtained with both simulators are highly reliable in relation to the human structures simulated in this study.

The homemade simulators also enable the trainee to learn how to adjust the ultrasound machine to obtain goog image, by mastering three basic knowledge: 1. Correct choice of ultrasound probe; 2. Adequacy of image gain; and 3. Adjust image depth.^7^

However, it is worth mentioning that, although these artisanal simulators have received positive assessments of participants in practical sessions about renal biopsy and venous access guided by ultrasonography in severa ultrasound courses for nephrologists, it will be important validate, in future studies, whether the training with these models will enable physician to perform both procedures in humans in a secure and accurate manner.

In short, the artisanal simulators described, inexpensive and easy and quick to assemble, allows the simulation of ultrasound-guided venous access and renal biopsy, and should be considered an alternative to the commercial phantoms available in the market.
